# Supplementary figures and images for: Tissue-specific transfer learning improves functional variant and therapeutic target discoveries in breast and prostate cancer
Source: PLoS Genet. 2026 May 6;22(5):e1012145. doi: 10.1371/journal.pgen.1012145 (PMC13175468; doi:10.1371/journal.pgen.1012145)

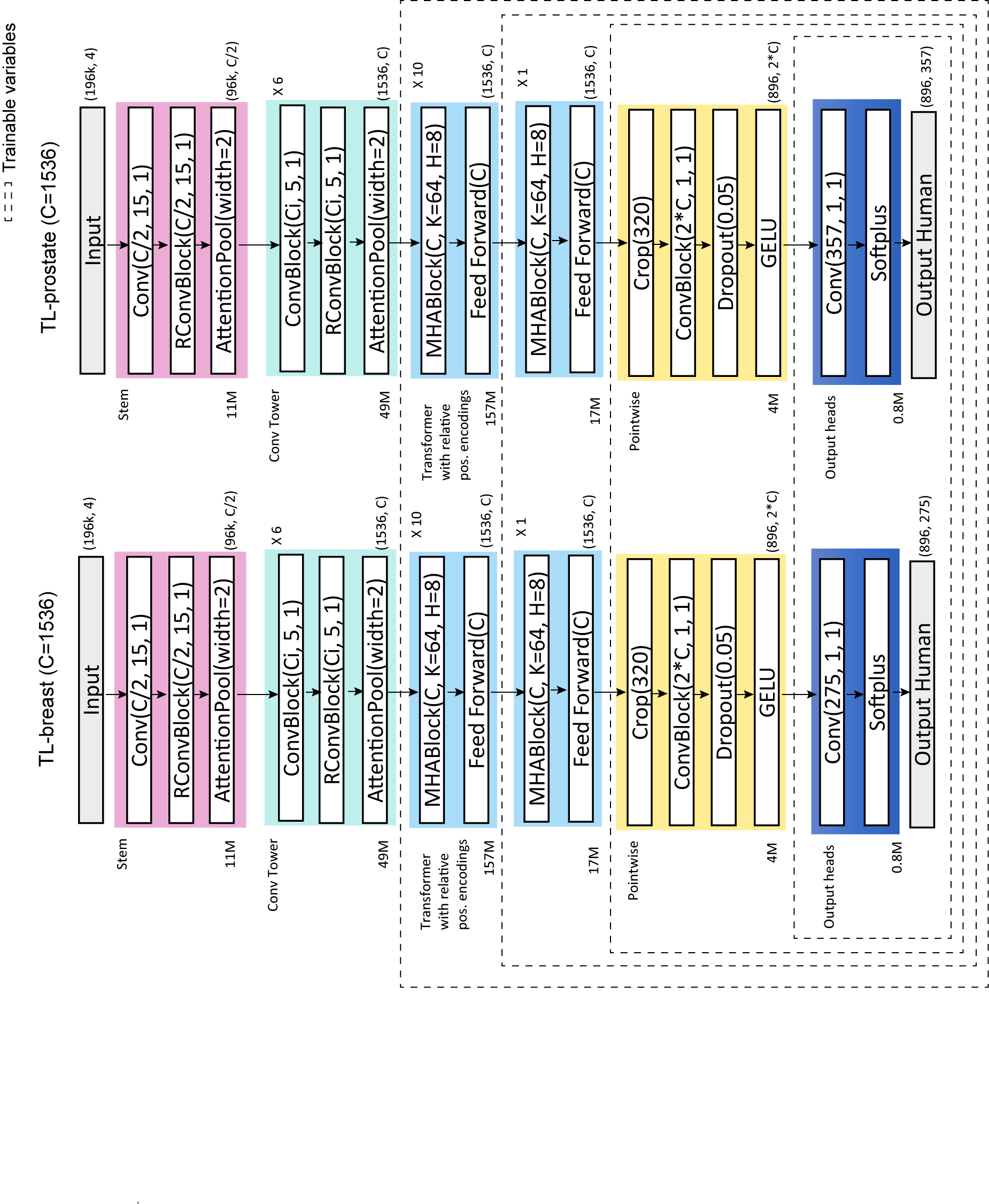

Supplement: S1 Fig — The TL model is segmented into five distinct blocks, each composed of multiple layers. The output shapes, excluding the batch dimensions, are denoted by tuples situated to the right of each block. ‘C’ denotes the number of channels, amounting to 1,536. The TL models is characterized by a single output head, fine-tuned to cater to its designated TF tracks. (TIF) [file pgen.1012145.s001.tif]

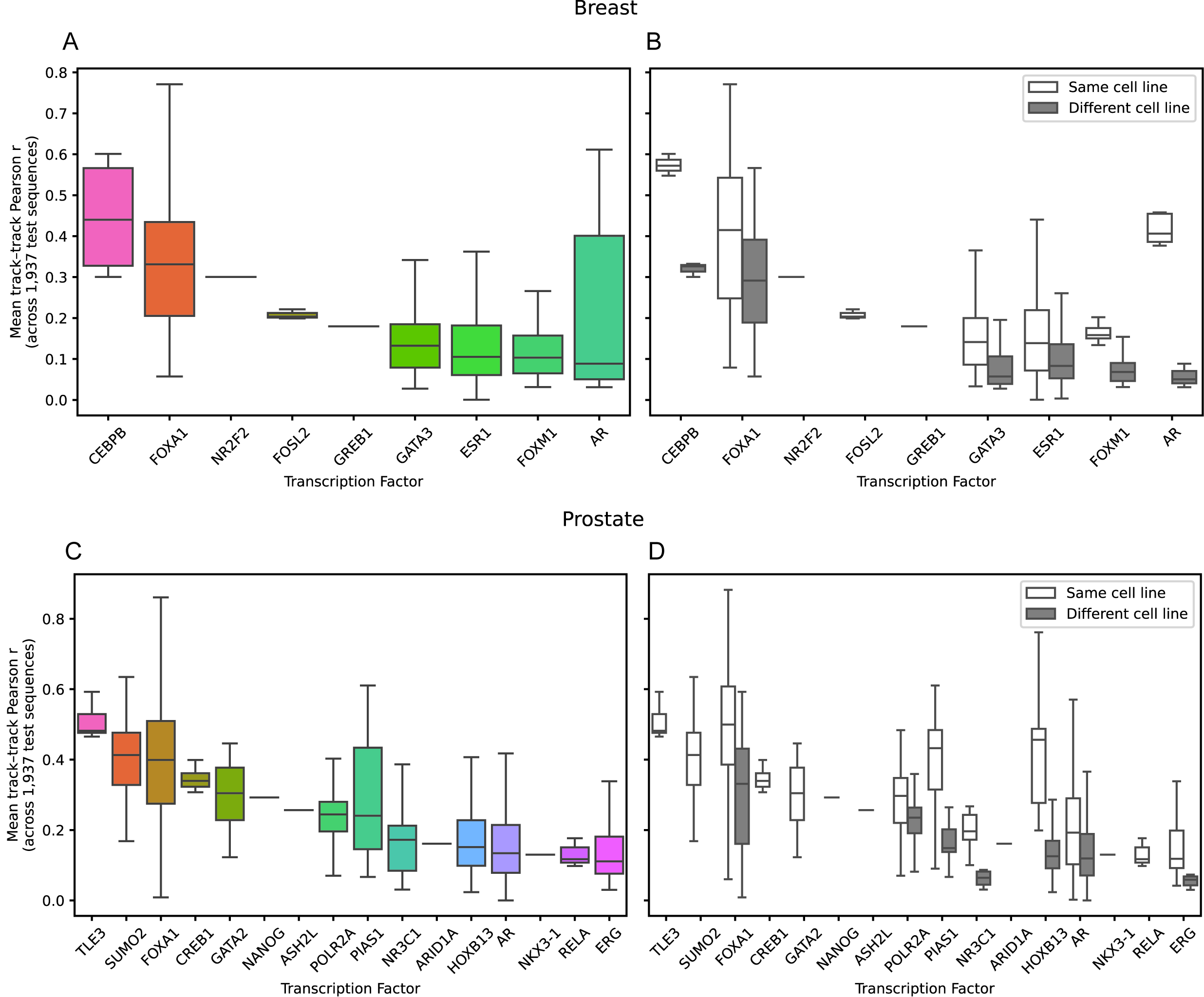

Supplement: S2 Fig — A) Track-track pairwise Pearson correlations across 1,937 held-out test sequences for TFs in breast tissue; B) Same as (A), stratified by cell line; C) Track-track pairwise Pearson correlations across 1,937 held-out test sequences for TFs in prostate tissue; D) Same as (C), stratified by cell line. (TIF) [file pgen.1012145.s002.tif]

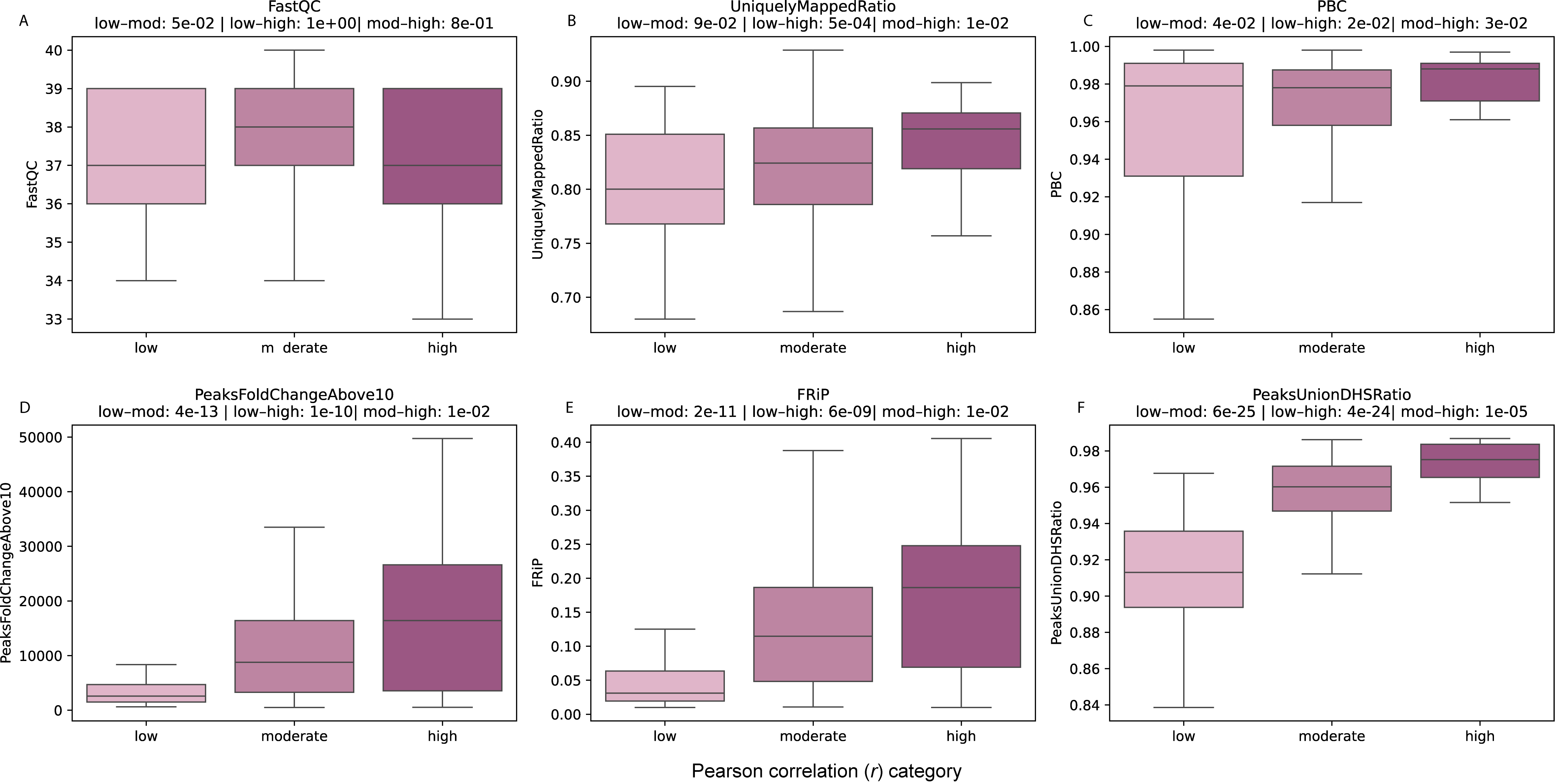

Supplement: S3 Fig — A) FastQC score, reflecting overall sequencing quality. B) Uniquely mapped read ratio, representing the proportion of reads that align uniquely to the reference genome. C) PCR bottleneck coefficient (PBC), measuring library complexity and PCR duplication bias. D) Peaks Fold Change over 10, indicating the proportion of peaks with at least ten-fold enrichment over background signal, reflecting peak strength. E) Fraction of reads in peaks (FRiP), quantifying signal enrichment within called peak regions. F) Union DNase I hypersensitive site (DHS) ratio, representing the fraction of reads overlapping open chromatin regions. (TIF) [file pgen.1012145.s003.tif]

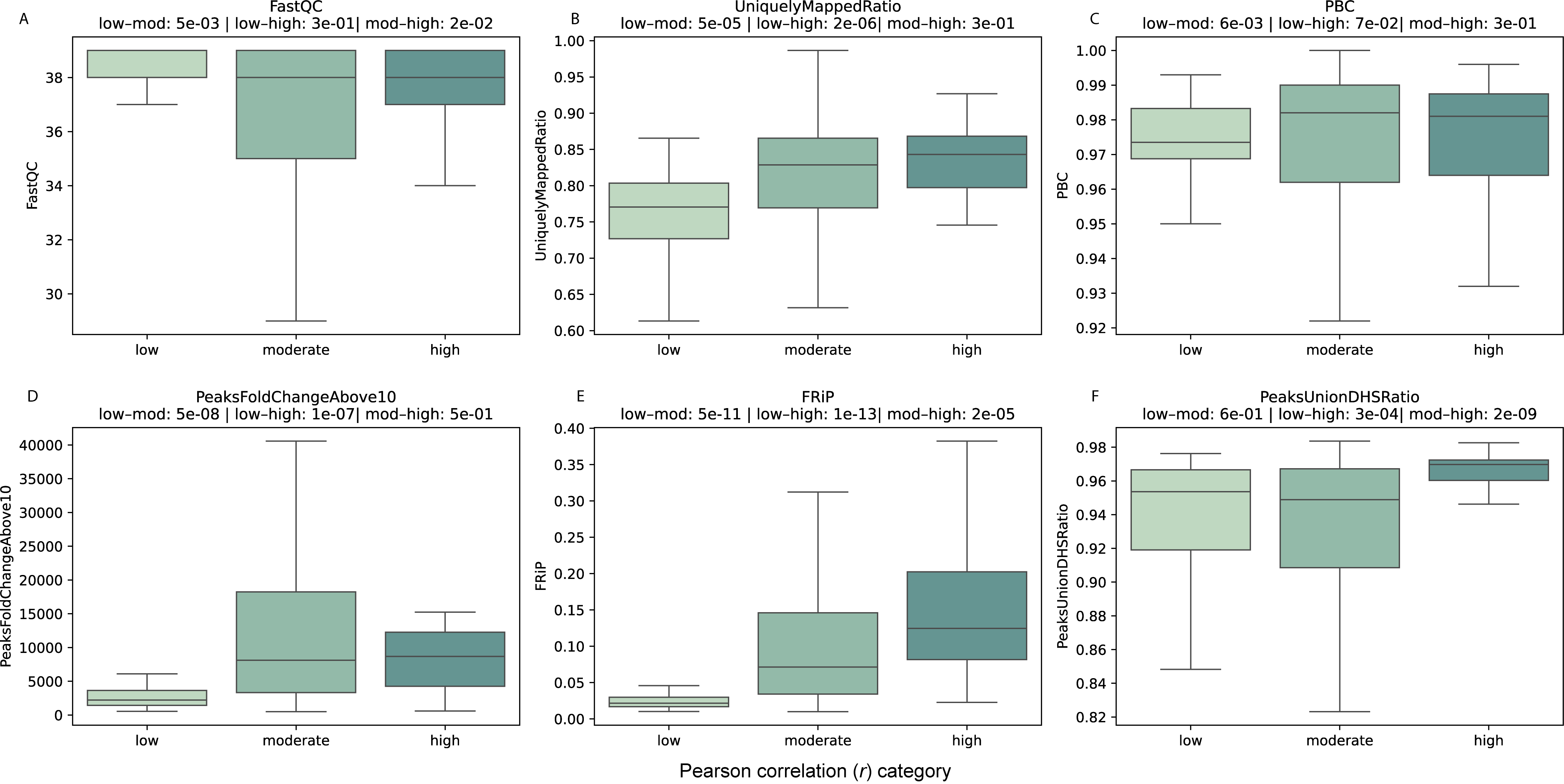

Supplement: S4 Fig — A) FastQC score. B) Uniquely mapped read ratio. C) PCR bottleneck coefficient (PBC). D) Peaks Fold Change over 10. E) Fraction of reads in peaks (FRiP). F) Union DNase I hypersensitive site (DHS) ratio. (TIF) [file pgen.1012145.s004.tif]

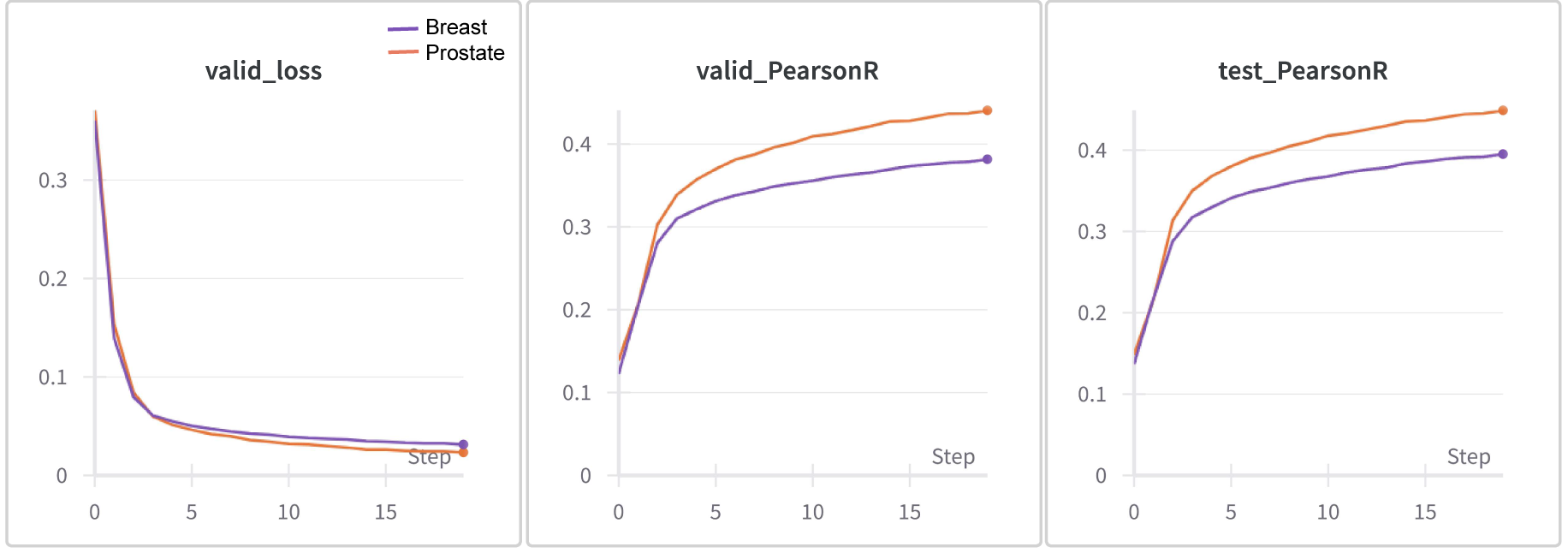

Supplement: S5 Fig — (TIF) [file pgen.1012145.s005.tif]

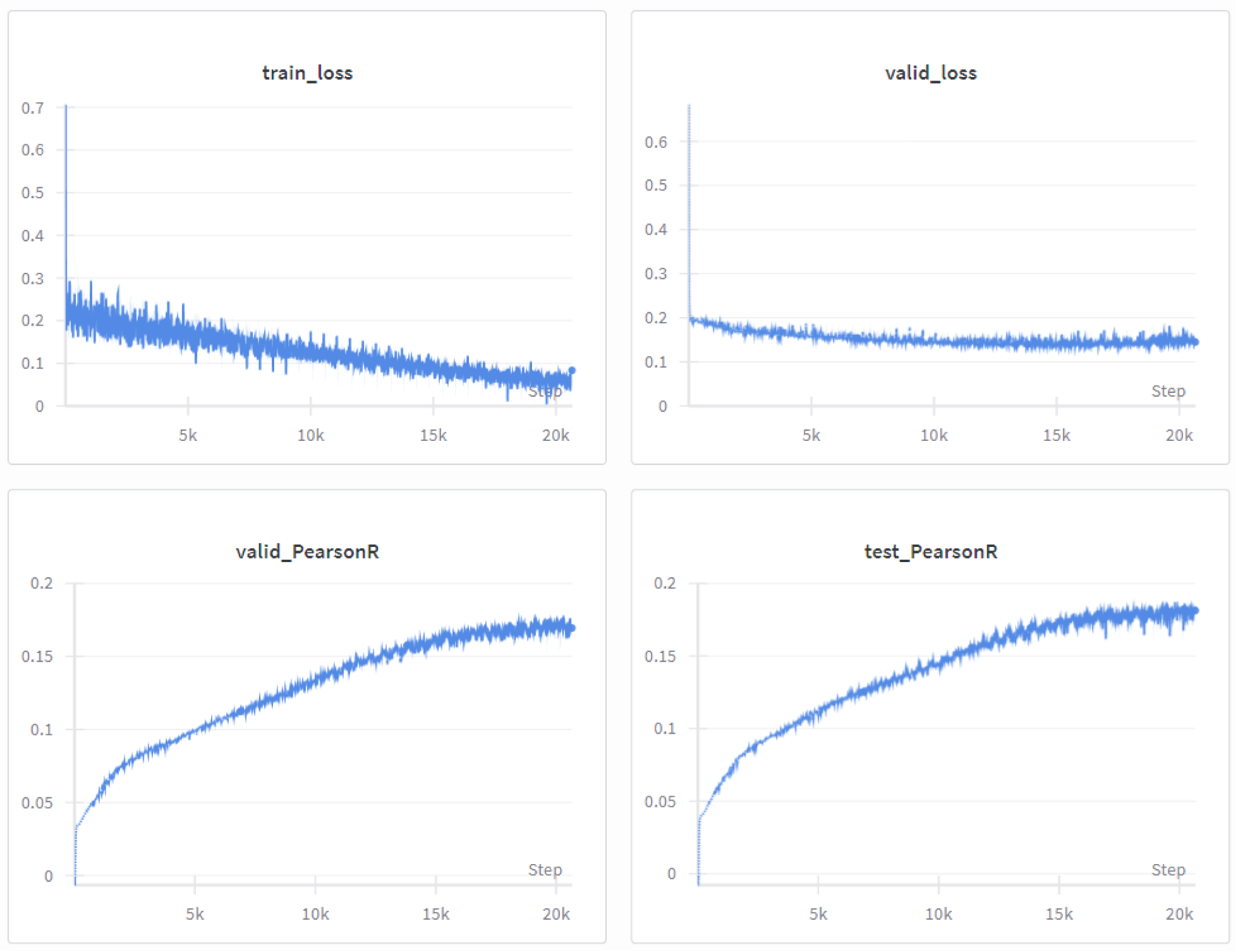

Supplement: S6 Fig — (TIF) [file pgen.1012145.s006.tif]
