# Supplementary material for: Tissue-specific transfer learning improves functional variant and therapeutic target discoveries in breast and prostate cancer
Source: PLoS Genet. 2026 May 6;22(5):e1012145. doi: 10.1371/journal.pgen.1012145 (PMC13175468; doi:10.1371/journal.pgen.1012145)
Supplement: Attachment — Submitted filename: Responses_Letter.pdf [file pgen.1012145.s024.pdf]

Reviewer #1: In this paper, the authors employed a transfer learning approach to tune Enformer into tissue-specific models and prioritize SNVs associated with breast and prostate cancers. Overall, the methodological framework is feasible, and the results are compelling. The proposed approach and findings are likely to provide valuable insights and tools for the human genetics and computational genomics communities. Here are some suggestions and comments for this work.

Thank you for the overall positive evaluation. We are grateful to your constructive comments and have conducted extensive works to address them. Please see our item-to-time response below.

R1.1 For transcription factors (TFs) in the TL-breast or TL-prostate models that overlap with Enformer's output tracks (or correspond to TFs in biologically similar tissues), could the authors **compare Enformer's predictions with those from the TL-breast/TL-prostate models using the same sequence inputs**, analogous to the setup shown in Figure 2? Such a comparison would provide a clear and direct evaluation of model performance prior to the SNV prioritization analysis in figure 3.

Responses: As suggested by the reviewer, we compared the outputs of TL models with those of the original Enformer model using the overlapping TFs and the same input DNA sequences. Only one TF, **POLR2A**, was shared between the TL-prostate model and Enformer. The TL-prostate model contains 42 POLR2A-associated tracks (**S2 Table**), whereas Enformer contains 4 corresponding tracks (**S5 Table**) from prostate. To reduce track-level heterogeneity, we averaged predictions across tracks within each model, yielding a vector of 896 values per model for each input DNA sequence. We then computed Pearson correlations between the TL-prostate and Enformer prediction vectors across 1,973 held-out sequences in test dataset. The two models showed a notable concordance, with a mean correlation of **0.55** (**S6 Table**). This comparison suggests that TL-prostate recapitulates much of Enformer's regulatory signal for POLR2A despite being fine-tuned on tissue-specific data. We have incorporated results and methods into the revised manuscript.

Results

*"To further evaluate consistency between the TL models and Enformer on matched inputs, we compared their predictions across 1,937 input DNA sequences from test datasets per transcription factor (**Materials and Methods**). POLR2A was the only TF shared between the two models, with 42 tracks in the TL-prostate model (**S2 Table**) and four corresponding tracks in Enformer (**S5 Table**). Using this TF, we observed a notable concordance between model outputs, with an average Pearson correlation of 0.55 (**S6 Table**), indicating that the TL-prostate model preserves much of Enformer's regulatory signal for POLR2A while incorporating tissue-specific refinements introduced during transfer learning." (page 7)*

Methods

"***Performance comparison of TL and Enformer models.*** *To evaluate the consistency between the TL models and the original Enformer model, we compared their predictions for overlapping TFs using identical input DNA sequences. For each tissue, we identified TFs present in both the TL model and Enformer. To reduce track-level heterogeneity, predictions were averaged across all tracks corresponding to the same TF within each model, yielding a vector of 896 output values per model for each input sequence. We calculated the Pearson correlations between the TL-model and Enformer prediction vectors across 1,973 DNA sequences from the held-out test dataset." (page 18)*

R1.2 To demonstrate the necessity and contribution of the knowledge learned from Enformer, it would be valuable to include a benchmark model trained de novo without leveraging any pretrained foundation model—for example, a simple CNN mapping DNA sequence directly to tissue-specific epigenomic features. Otherwise, if a basic CNN achieves comparable performance, the advantage of utilizing Enformer as the foundation model would be questionable. Evaluating this comparison on a few representative transcription factors should be sufficient to illustrate the point.

Responses: To directly assess the necessity and contribution of pretrained regulatory knowledge learned by Enformer, we implemented a simple convolutional neural network (CNN) that maps raw DNA sequence directly to TF ChIP–seq outputs, without leveraging any pretrained foundation model.

To ensure a fair comparison, the CNN was designed to match the TL models in both input and output dimensionality. The model consists of four convolutional layers with 128, 256, 512, and 512 channels, respectively, each using a kernel size of 15 and stride sizes of 2, 4, 4, and 4. Input sequences of 196,608 bp were symmetrically cropped by 40,960 bp at each end to yield 114,688 bp, which were then progressively down sampled as follows: 57,344 × 128 →14,336 × 256 → 3,584 × 512 → 896 × 512. A final convolutional head projected the resulting 896 × 512 representation to 896 × k, where k denotes the number of TF ChIP–seq tracks. We utilized the breast TF ChIP–seq tracks as an example, and k is equal to 275.

The CNN was trained entirely from scratch using the same optimization settings as the TL models (Adam optimizer, learning rate 1×10^{-4}, batch size 64, and Poisson negative log-likelihood loss). Training was performed on an NVIDIA Tesla V100 GPU. After three days' training, the CNN achieved Pearson correlation coefficients of 0.18 on the test datasets (**S4 Fig**), respectively, substantially lower than those observed for the TL-breast models (**S3 Fig**). Although extending the training duration could potentially improve CNN performance, TL models stopped training within three days. Therefore, to ensure a fair comparison in terms of training time, CNN training was likewise restricted to a three-day period. To further assess the functional relevance of variants prioritized by the CNN, we evaluated enhancer enrichment among the top 1M, 1.5M, and 2M ranked SNPs. Across all subsets, the CNN consistently exhibited reduced enhancer enrichment compared with the TL-Breast models (CNN vs. TL-breast: 1M subset

(7.5% vs. 13.3%), 1.5M subset (7.3% vs. 11.7%) and 2M subset (7.1% vs. 10.6%)), indicating inferior prioritization of regulatory variants.

Together, these results demonstrate that training a simple CNN from scratch on TF-specific ChIP–seq data is insufficient to recover robust regulatory representations, particularly for capturing long-range cis-regulatory effects. In contrast, TL models benefit from Enformer's pretrained multi-scale regulatory knowledge, leading to substantially improved predictive performance and downstream variant prioritization. Moreover, training the original Enformer model required large-scale computational resources, including 64 TPU v3 cores with a batch size of 64 (one sample per core) for approximately three days. Access to such large number of TPU/GPU is not feasible for many research groups in including us. In this context, transfer learning provides a practical and efficient alternative, substantially reducing computational cost and training time while preserving the advantages conferred by pretrained regulatory representations.

We added the following content to **Discussion** section and **Method and Material** section in the revised manuscript.

Discussion

"A key novelty of our framework lies in its application of transfer learning. Functional annotation using epigenomic data from non-target tissues can introduce signals unrelated to the disease context, creating a fundamental trade-off in multi-scale modeling: excessively heterogeneous data may dilute disease-relevant biology, whereas overly narrow models limit generalizability. Our transfer learning strategy addresses this challenge through a two-step design. First, a foundation model is trained on diverse multi-omic data to capture broad regulatory features. Second, this general regulatory model is redirected toward the appropriate biological context by fine-tuning with tissue- or disease-specific TF ChIP-seq profiles. Empirically, we also demonstrate the necessity of transfer learning. A simple convolutional neural network trained from scratch on breast TF ChIP-seq data failed to learn long-range regulatory dependencies and performed substantially worse than the TL-breast model on held-out data (Pearson r = 0.18 vs. 0.39; ***S3 Fig, S4 Fig****). Functional enrichment was similarly weaker for the CNN across the top 1M, 1.5M, and 2M SNP subsets (CNN vs. TL-breast: 1M subset (7.5% vs. 13.3%), 1.5M subset (7.3% vs. 11.7%) and 2M subset (7.1% vs. 10.6%)), underscoring its limited ability to capture biologically meaningful regulatory signals. Moreover, transfer learning also confers substantial computational advantages: fine-tuning of TLs was completed within three days on a single NVIDIA A100 GPU, whereas training Enformer from scratch requires 64 TPU v3 cores for a similar duration. Thus, transfer learning offers a practical and efficient strategy for leveraging rich regulatory knowledge embedded in pretrained foundational models while adapting them to disease-relevant cellular contexts." (page 13)*

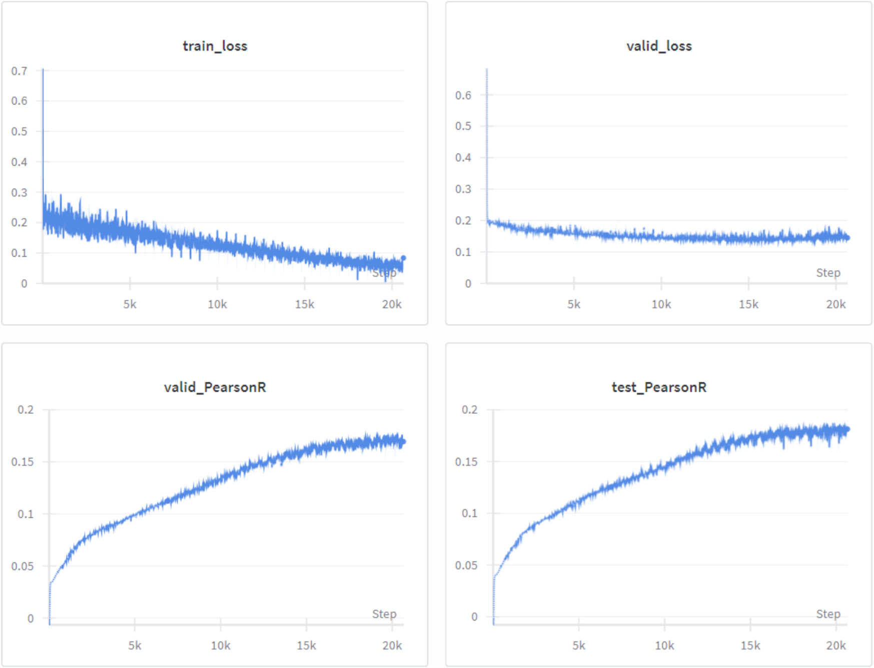

S4 Fig: Loss and performance of the baseline CNN model trained on breast TF ChIP-seq data.

Methods and Materials

*"**Baseline convolutional neural network trained from scratch.** To benchmark the necessity of pretrained regulatory knowledge from Enformer, we constructed a baseline convolutional neural network trained from scratch to predict TF ChIP–seq signals directly from DNA sequence. The CNN architecture consisted of four convolutional layers (128, 256, 512, and 512 channels; kernel size = 15; strides = 2, 4, 4, and 4), followed by a final convolutional projection layer. Input sequences of 196,608 bp were symmetrically cropped to 114,688 bp and progressively down sampled to yield an output tensor of 896 bins × k tracks, where k is the number of TF ChIP–seq datasets (k = 275 for breast TFs). The CNN was trained de novo using the same optimization settings as the TL models (Adam optimizer, learning rate = 1×10^{-4}, batch size = 64, Poisson negative log-likelihood loss) on an NVIDIA Tesla V100 GPU. To ensure a fair comparison, training time was capped at three days, matching the training time of TL models. Model performance was evaluated using Pearson correlation between predicted and observed ChIP–seq tracks on held-out validation and test sets." (page 18)*

R1.3 For Figures 4C–E, could the authors include results from conventional TWAS analyses as a baseline comparison? Although some of these results are provided in the supplementary tables, researchers would likely be interested in directly visualizing how the SNVs derived from the TL models compare with results from standard TWAS approaches that use a larger set of SNPs.

Responses: As recommended by the reviewer, we have added the proportion of genes classified as druggable targets and DisGeNET-associated genes to **Figures 4C–4E** (indicated by grey dashed lines). The corresponding numerical values are provided in **S10 Table**. Overall, genes identified by the TL models exhibited higher rates of druggable and DisGeNET targets compared to the baseline model. The revised Figure 4 is included here:

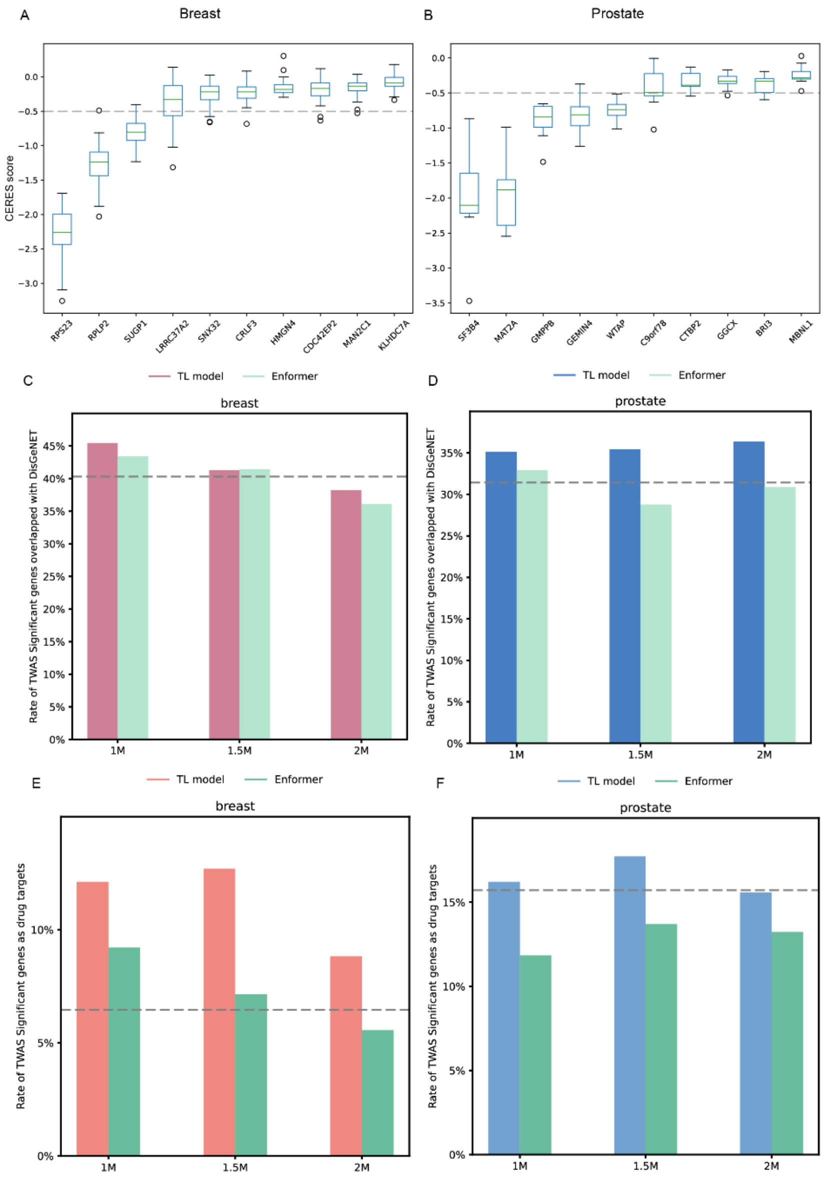

Fig 4: Biological and therapeutic relevance for TWAS significant genes. A) Boxplots depicting top 10 genes dependency (CERES) scores derived from breast cancer (48 cell lines) CRISPR-Cas9 experiments (DepMap 23Q4) for TL-breast model three SNVs subsets. The dashed line represents a suggested CERES value cutoff of −0.5, below which indicates a higher dependency of cell proliferation on the gene. **B)** The same as A, but from prostate cancer (9 cell lines) for genes identified by the TL-prostate model. **C)** Bars plot to show percentage of TWAS significant genes as drugs targets, utilizing TL-breast or Enformer-prioritized subsets of SNVs. **D)** The same as **C)** but for TWAS results using TL-prostate or Enformer-prioritized subsets of SNVs. **E**) DisGeNET enrichment rate (percentage of TWAS-significant genes found in DisGeNET) for TL-breast and Enformer models across the three SNV subsets. **F)** The same as **E)** but for prostate cancer. Grey dashed line refers to the baseline model result in **C-E**.

R1.4 Following the previous point, it would be helpful if the authors could include a discussion on the advantages and limitations of selecting SNVs from an external model when constructing TWAS linear models for gene expression prediction, compared to using the standard TWAS approach. Such a discussion could clarify the potential benefits of leveraging TL-derived SNVs, as well as any trade-offs or biases introduced by this selection strategy.

Responses: We appreciate the reviewer's insightful comments and have incorporated a detailed discussion of the advantages and limitations of this approach into the revised **Discussion** section.

Discussion:

"*Another key strength of our framework is that it allows us to prioritize variants with functional relevance synergically. Unlike traditional TWAS approaches that select SNVs solely based on genotype–expression correlations, our method restricts predictors to SNVs enriched for regulatory activity, thereby enhancing statistical power and improving interpretability by anchoring TWAS associations to experimentally informed regulatory elements. Nevertheless, the use of TL-derived SNVs mainly dependent on the completeness and accuracy of the external model, which may introduce tissue- or assay-specific. Although our enhancer-enrichment analyses support the functional relevance of prioritized variants, complementary approaches will be needed to more fully characterize their regulatory architecture and ensure that model-derived SNVs capture the full spectrum of biologically meaningful mechanisms.*" *(page 14)*

As a relevant example, but a different case, imagine a strategy in which half of the GWAS data are used to generate summary statistics for prioritizing the SNPs included in the TWAS model, and the remaining half are used for the gene–phenotype association test. What could happen?

Responses: The last step of gene-phenotype association in our analysis was associated between genetically predictive model (GReX model trained between gene expression genetic variates) and cancer GWAS summary statistics. For the success of association tests, the GReX model and the GWAS summary statistics must share the same SNPs. If half of the GWAS were used to prioritize SNPs for inclusion in the TWAS model and the remaining half were used for gene–phenotype association, the SNP sets between the GReX model and the association data would not overlap. As such, the proposed comparison is not feasible for us.

R1.5 In figure S2, the TL models seem not fully converged without reaching a plateau. Maybe the authors can elongate the training a bit. This is a minor and trivial issue.

Responses: We thank the reviewer for this suggestion. Although the training curves do not exhibit a strict plateau, training was intentionally halted using an early-stopping strategy, defined

as an improvement in validation loss of <0.001 for five consecutive evaluation steps (**Materials and Methods**). This strategy was adopted to prevent over-optimization during transfer learning, as excessively prolonged training risks overwriting or distorting regulatory representations inherited from the pretrained Enformer model. Further extending training could therefore alter the model's learned features and potentially confound downstream analyses. For these reasons, we retained the current training configuration.

R1.6 It would be helpful to provide the complete list of transcription factors (TFs) associated with the diseases of interest, such as breast or prostate cancer, along with the subset of TFs used in the TF model training. Ideally, all relevant TFs would be included in the training; however, data availability may be a limiting factor. Providing these lists would allow users to better understand the potential limitations of the model and its coverage of disease-relevant regulatory factors.

Responses: As requested by the reviewer, we have provided the list of transcription factors (TFs) previously shown to have notable associations with breast and prostate cancer using a generalized linear mixed model [PMID: 34518541] (**S15 Table**). In this work, we have incorporated **82% (18/22)** of the breast cancer risk TFs and all 17 prostate cancer risk TFs, contingent upon the availability of high-quality TF ChIP-seq datasets in the Cistrome database.

R1.7 The x-axis labels in Figure 3A–B (e.g., "1M") should be centered between the two columns, as shown in Figure 4C–D.

Responses: Based on this comment, we modified the labels accordingly.

Reviewer #2: Li, et al. adapt the Enformer model for variant effect prediction using transfer learning in order to focus on transcription factor tracks from breast and prostate cancer cell lines. They then evaluate the predicted variant effects from this new model compared to the original Enformer model.

Thank you for the summary of our works. And thank you so much for the helpful comments based on which we have substantially improved the manuscript. Please find our item-to-item response below.

Major comments

R2.1 Clarify what training data was used.

The authors state:

"For breast cancer, we obtained tracks for 18 TFs … yielding a total of 275 tracks"

"For prostate cancer, we selected 17 TFs … totaling 357 tracks"

The Supplementary Tables indicate multiple cell lines were used for each TF, but it looks like many of the tracks a biological or technical replicates. How many biologically distinct tracks were used and how were replicates handled?

Responses: We thank the reviewer for this insightful question. We acknowledge that multiple cell lines were used for each TF. Our intention in assembling the training data was to include as many high-quality TF ChIP-seq datasets as possible, allowing the deep learning model to learn consistent TF-binding patterns across experiments while accommodating variability arising from differences in cell lines, experimental conditions, and batch effects.

As Cistrome aggregates experiments from multiple laboratories and conditions, tracks for the same TF may represent a mixture of biological replicates, technical replicates, and data generated in distinct cell lines. To address this, we have applied the following strategies: 1) Distinct cell lines: When a TF had ChIP-seq data available across multiple cell lines, each TF–cell line combination was treated as a separate biological context and retained as an independent training track. 2) Biological replicates: Independent experiments profiling the same TF in the same cell line were retained individually, as they capture genuine biological variability and can improve model robustness. 3) Technical replicates: These are not explicitly distinguishable within Cistrome metadata. However, because TL model training treats each track independently and does not require balancing replicate counts, the presence of technical replicates does not adversely affect model performance. We added these details into **Materials and Methods** section.

Materials and Methods

"Each TF–cell line combination was treated as a distinct biological context and retained as an independent training track, and independent experiments profiling the same TF in the same cell line were also retained individually. Technical replicates could not be explicitly identified from Cistrome metadata." (page 16)

In Figure 2, the performance often varies widely across many tracks for the same TF. Is this biological or technical?

Responses: To evaluate whether the variability in **Fig 2** reflects biological or technical sources, we quantified concordance among ChIP–seq tracks corresponding to the same TF using the original ChIP–seq signal intensities across the same 1,937 test sequences displayed in **Fig 2**. For each TF with multiple tracks, we computed pairwise correlations per sequence and averaged these values across sequences (**S3–S4 Table**). Our analysis revealed substantial heterogeneity among tracks for the same TF in both breast and prostate datasets (**S2A, S2C, Fig**). Importantly, much of this variability is biological as tracks generated from different cell lines showed the greatest divergence, whereas tracks derived from the same cell line were considerably more concordant (**S2B, S2D, Fig**). This pattern indicates that cell-line context is a major determinant of track-level differences. Technical variability may also contribute. However, Cistrome metadata do not reliably distinguish technical replicates, preventing a systematic evaluation of their impact. Overall, the range of performance across tracks for the same TF is consistent with the underlying biological heterogeneity of publicly available ChIP–seq sources. We have added details to **Results, Materials and Methods** and **Discussion** section.

Results

*"Among these ChIP-seq tracks, pairwise correlation analyses (**Materials and Methods**) showed presence of substantial biological heterogeneity (**S3-S4 Table**), with tracks derived from the same cell line exhibiting significantly higher concordance than tracks originating from different cell lines (**S2 Fig**)." (page 5)*

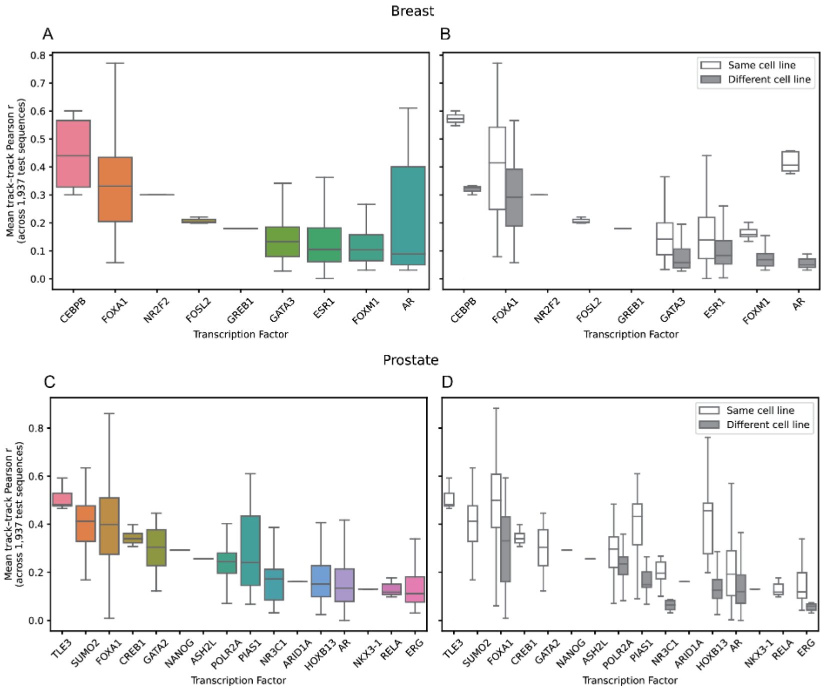

S2 Fig: Track-track Pearson correlations. A) Track-track pairwise Pearson correlations across 1,937 held-out test sequences for TFs in breast tissue; **B**) Same as (A), stratified by cell line; **C**) Track-track pairwise Pearson correlations across 1,937 held-out test sequences for TFs in prostate tissue; **D**) Same as (C), stratified by cell line.

Materials and Methods

*“**Pairwise correlation of ChIP–seq tracks.** To assess biological concordance among ChIP–seq tracks corresponding to the same TF, we collected all available tracks for each TF and quantified their similarity using the original ChIP–seq signal intensities. Pairwise correlations were computed between all track pairs based on 1,937 sequences targets from the held-out test dataset. For each track pair, Pearson correlation coefficients were calculated on a per-sequence basis and subsequently averaged across 1,937 sequences to generate an overall measure of track-level concordance for each TF.” (page 16)*

Discussion

“The TF ChIP-seq tracks used for transfer learning include mixtures of biological and technical replicates, contributing to heterogeneity across tracks. Tracks from the same cell line generally showed more consistent behavior, whereas tracks from different cell lines displayed markedly greater divergence. Future efforts to disentangle replicate structure may further improve the characterization of TF-specific regulatory signals for model training.” (page 15)

R2.2 Demonstrate contribution of transfer learning

The authors seek to demonstrate the value of adapting a pretrained Enformer model using transfer learning. While they demonstrate that training on their specific cancer tracks is useful, they don’t directly demonstrate the benefit of transfer learning compared to training a new model without the pretraining step. Does using transfer learning instead of a new set of parameters just reduce training time or meaningfully increase predictive performance?

Responses: We thank the reviewer for this question. To directly evaluate whether TL with a pretrained Enformer backbone provides benefits beyond reduced training time, we benchmarked the performance of TL-model with a simple convolutional neural network (CNN). As a reference, training the original Enformer model required substantial computational resources, including 64 TPU v3 cores with a batch size of 64 (one sample per core) for approximately three days. Because access to such large-scale TPU or GPU resources is unavailable for most research groups including us, we selected a computationally feasible CNN model trained entirely from scratch.

To ensure a fair comparison, the CNN was designed to match the TL models in both input and output dimensionality. The model consists of four convolutional layers with 128, 256, 512, and 512 channels, respectively, each using a kernel size of 15 and stride sizes of 2, 4, 4, and 4. Input sequences of 196,608 bp were symmetrically cropped by 40,960 bp at each end to yield 114,688 bp, which were then progressively down sampled as follows: 57,344 × 128 →14,336 ×

256 → 3,584 × 512 → 896 × 512. A final convolutional head projected the resulting 896 × 512 representation to 896 × k, where k denotes the number of TF ChIP–seq tracks. We utilized the breast TF ChIP–seq tracks as an example, and k is equal to 275.

The CNN was trained entirely from scratch using the same optimization settings as the TL models (Adam optimizer, learning rate 1×10^{-4}, batch size 64, and Poisson negative log-likelihood loss). Training was performed on an NVIDIA Tesla V100 GPU. After three days' training, the CNN achieved Pearson correlation coefficients of 0.18 on the test datasets (**S4 Fig**), respectively, substantially lower than those observed for the TL-breast models (**S3 Fig**). Although extending the training duration could potentially improve CNN performance, TL models stopped training within three days. Therefore, to ensure a fair comparison in terms of training time, CNN training was likewise restricted to a three-day period. To further assess the functional relevance of variants prioritized by the CNN, we evaluated enhancer enrichment among the top 1M, 1.5M, and 2M ranked SNPs. Across all subsets, the CNN consistently exhibited reduced enhancer enrichment compared with the TL-Breast models (CNN vs. TL-breast: 1M subset (7.5% vs. 13.3%), 1.5M subset (7.3% vs. 11.7%) and 2M subset (7.1% vs. 10.6%)), indicating inferior prioritization of regulatory variants.

Together, these results demonstrate that training a simple CNN from scratch on TF-specific ChIP–seq data is insufficient to recover robust regulatory representations, particularly for capturing long-range cis-regulatory effects. In contrast, TL models benefit from Enformer's pretrained multi-scale regulatory knowledge, leading to substantially improved predictive performance and downstream variant prioritization. Moreover, training the original Enformer model required large-scale computational resources, including 64 TPU v3 cores with a batch size of 64 (one sample per core) for approximately three days. Access to such large number of TPU/GPU is not feasible for many research groups in including us. In this context, transfer learning provides a practical and efficient alternative, substantially reducing computational cost and training time while preserving the advantages conferred by pretrained regulatory representations.

We added the following content to **Discussion** section and **Method and Material** section in the revised manuscript.

Discussion

"A key novelty of our framework lies in its application of transfer learning. Functional annotation using epigenomic data from non-target tissues can introduce signals unrelated to the disease context, creating a fundamental trade-off in multi-scale modeling: excessively heterogeneous data may dilute disease-relevant biology, whereas overly narrow models limit generalizability. Our transfer learning strategy addresses this challenge through a two-step design. First, a foundation model is trained on diverse multi-omic data to capture broad regulatory features. Second, this general regulatory model is redirected toward the appropriate biological context by fine-tuning with tissue- or disease-specific TF ChIP-seq profiles. Empirically, we also demonstrate the necessity of transfer learning. A simple convolutional neural network trained from scratch on breast TF ChIP-seq data failed to learn long-range regulatory dependencies

and performed substantially worse than the TL-breast model on held-out data (Pearson r = 0.18 vs. 0.39; ***S3 Fig, S4 Fig****). Functional enrichment was similarly weaker for the CNN across the top 1M, 1.5M, and 2M SNP subsets (CNN vs. TL-breast: 1M subset (7.5% vs. 13.3%), 1.5M subset (7.3% vs. 11.7%) and 2M subset (7.1% vs. 10.6%)), underscoring its limited ability to capture biologically meaningful regulatory signals. Moreover, transfer learning also confers substantial computational advantages: fine-tuning of TLs was completed within three days on a single NVIDIA A100 GPU, whereas training Enformer from scratch requires 64 TPU v3 cores for a similar duration. Thus, transfer learning offers a practical and efficient strategy for leveraging rich regulatory knowledge embedded in pretrained foundational models while adapting them to disease-relevant cellular contexts." (page 13)*

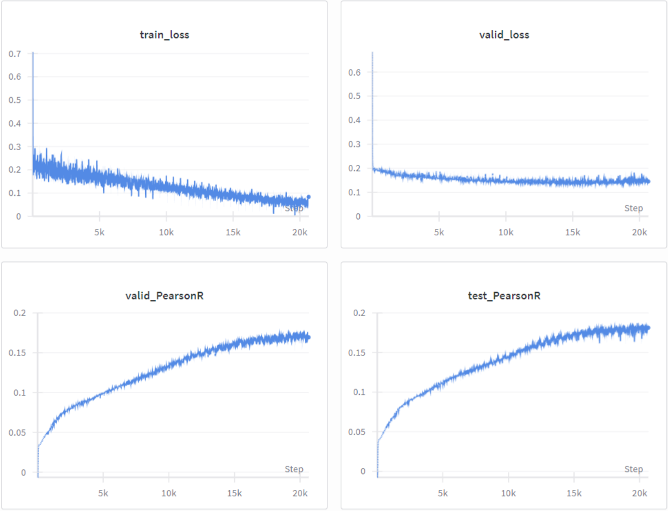

S4 Fig: Loss and performance of the baseline CNN model trained on breast TF ChIP-seq data.

Methods and Materials

*"**Baseline convolutional neural network trained from scratch.** To benchmark the necessity of pretrained regulatory knowledge from Enformer, we constructed a baseline convolutional neural network trained from scratch to predict TF ChIP–seq signals directly from DNA sequence. The CNN architecture consisted of four convolutional layers (128, 256, 512, and 512 channels; kernel size = 15; strides = 2, 4, 4, and 4), followed by a final convolutional projection layer. Input sequences of 196,608 bp were symmetrically cropped to 114,688 bp and progressively down sampled to yield an output tensor of 896 bins × k tracks, where k is the number of TF ChIP–seq datasets (k = 275 for breast TFs). The CNN was trained de novo using the same optimization settings as the TL models (Adam optimizer, learning rate = 1×10^{-4}, batch size = 64, Poisson negative log-likelihood loss) on an NVIDIA Tesla V100 GPU. To ensure a fair comparison, training time was capped at three days, matching the training time of TL models. Model performance was evaluated using Pearson correlation between predicted and observed ChIP–seq tracks on held-out validation and test sets." (page 18)*

Reviewer #3: This paper adapted and fine-tuned existing deep learning tool Enformer to breast and prostate cancer using 275 and 357 tissue-specific transcription factor (TF) ChIP–seq tracks. Further application of the fine-tuned Enformer include tissue-specific prioritization of variants using these variants for improving TWAS. It is claimed the proposed approach enhances regulatory variant prioritization and improve susceptibility gene discovery in a tissue-specific manner. I have some comments as follows,

Thank you for the summary of our works. And thank you so much for the helpful comments based on which we have substantially improved the manuscript. Please find our item-to-item response below.

R3.1 As so many TFs are involved in calculating the tCRAs for variant prioritization. However, the formula on line 400 only indicate one variant and one TF. Please clarify how the variant score is calculated considering so many TFs are collected and for each TF, there are multiple biological replicates (tracks). Please formularize these in a more rigorous way.

Responses: We apologize for any confusion caused by the mathematical formula. We define the tissue-based cis-regulatory activity (**tCRA**) as the absolute mean difference between predicted signals for the alternative and reference alleles, averaged across all tracks. Although many tracks reflect biological replicates, they still exhibit measurable heterogeneity, even within the same cell line, and substantially greater divergence across different cell lines (**S2 Fig**). Given this variability, treating each track as an independent output is reasonable. We included the revised formula in **Materials and Methods** section.

Materials and Methods

"

$$tCRA_i = \frac{1}{k}\sum_{j=1}^{k} \left|Pred_{i,j}^{alt} - Pred_{i,j}^{ref}\right| \quad (2)$$

where, $tCRA_i$ represents the regulatory activity of the i-th SNV, defined as the absolute mean difference between the predicted values for the alternative allele ($Pred_{i,j}^{alt}$) and reference allele ($Pred_{i,j}^{ref}$) across all tracks (j)." (page 19)

R3.2 More formula for loss function for TF-breast and TF-prostate are needed. Only mention "The model was trained to predict ChIP-seq signals using a Poisson negative log-likelihood loss function is not enough." A follow-up question is, as processed ChIP-seq signals are not count data, why use Poisson distribution?

Responses: ChIP-seq signals quantify TF binding across the genome, where non-zero values indicate TF occupancy and zeros indicate absence of binding. As a result, ChIP-seq data are inherently sparse: most genomic positions contain values near zero, while only a small fraction exhibits elevated signal intensities corresponding to true TF-binding events. The Poisson negative log-likelihood loss is therefore appropriate, as it effectively captures the dynamic range of binding signals, and naturally accommodates the large number of zero-valued positions. This allows the model to learn TF binding patterns more accurately than alternative loss formulations. Additionally, this loss function (implemented as tf.keras.losses.poisson() in tensorflow) is used in the original Enformer model and was incorporated into our training framework because it is well suited for the statistical properties of ChIP-seq data. As suggested by the reviewer, we have added the detailed Poisson negative log-likelihood loss formula to the **Materials and Methods** section.

Materials and Methods

"

$$L_{Poisson}(T'(d;\ \Theta'), f(C)\ ) = T'(d;\ \Theta') - f(C)\ \cdot log\big(T'(d;\ \Theta')\big)(1)$$

Here, $d \in D'$ denotes an input DNA sequence fragment, and $T'(d;\ \Theta')$ is the predicted ChIP-seq signal produced by the TL-model T' with trainable parameters Θ'. The observed target $f(C)$ represents the preprocessed ChIP-seq signal corresponding to the same genomic region as d, obtained via a preprocessing function $f(\cdot)$ applied to the raw ChIP-seq data C, This loss function is used to fine-tune the target parameters Θ' of in the model T'." (page 18)

R3.3 One key concern is the prediction performance is very low (Fig2) but the downstream analysis of variant effect is based on the difference of accurate prediction between ref and alt, which make the variant effect analysis less reliable.

Responses: Thank you for this insightful comment. We respectfully disagree that the model performance in Fig. 2 should be interpreted as "very low," as average correlations around ~0.4 are well within the expected range for TF ChIP-seq prediction and align with previously published deep-learning models.

In **Fig 2**, we observed substantial variability in correlation performance across TFs. Some TFs achieved higher prediction accuracy, whereas others showed more modest correlations. This variation likely reflects the underlying biological heterogeneity of the ChIP-seq tracks: TFs with abundant and well-defined binding sites are easier for the model to learn, whereas TFs with sparse, context-dependent, or low-signal binding profiles are inherently more difficult to predict. In the breast dataset, 14 TFs exhibited correlations above the average (0.42), and in the prostate dataset, 10 TFs showed median correlations below the average (0.47), indicating that many TFs achieved modest but meaningful concordance.

Importantly, our performance is consistent with that reported for state-of-the-art models. Although Enformer does not report per-TF prediction correlations, another prior model Basenji provided TF ChIP-seq prediction performance in its S3 Fig [PMID:

32687525](https://doi.org/10.1371/journal.pcbi.1008050.s003). We found that our results fall within the performance range previously reported for Basenji, spanning a wide range (0.379 for REST to 0.78 for POLR2A). For TFs represented in both the TL models and Basenji, the predictive performance is also comparable. For example, for **JUND** in the TL-breast model, the mean correlation was **0.58**, compared with Basenji's **0.54** (row 6, column 2). For **POLR2A** in the TL-prostate model, our mean correlation was **0.78**, whereas Basenji reported **0.659** (row 4, column 2). Consistent with prior observations from both Enformer and Basenji, TF-binding predictions generally exhibit slightly lower correlations than DNase-seq or histone modification ChIP-seq (H3K4me3, H3K27ac, etc.), yet average correlations around ~0.4 or higher are commonly reported. Overall, the performance variation we observe is expected given the biological diversity of TF binding and aligns well with established benchmarks in the field.
