# Supplementary material for: Tissue-specific transfer learning improves functional variant and therapeutic target discoveries in breast and prostate cancer
Source: PLoS Genet. 2026 May 6;22(5):e1012145. doi: 10.1371/journal.pgen.1012145 (PMC13175468; doi:10.1371/journal.pgen.1012145)
Supplement: Attachment — Submitted filename: Reviewer_comments.docx [file pgen.1012145.s022.docx]

In this paper, the authors employed a transfer learning approach to tune Enformer into tissue-specific models and prioritize SNVs associated with breast and prostate cancers. Overall, the methodological framework is feasible, and the results are compelling. The proposed approach and findings are likely to provide valuable insights and tools for the human genetics and computational genomics communities. Here are some suggestions and comments for this work.

1. For transcription factors (TFs) in the TL-breast or TL-prostate models that overlap with Enformer’s output tracks (or correspond to TFs in biologically similar tissues), could the authors compare Enformer’s predictions with those from the TL-breast/TL-prostate models using the same sequence inputs, analogous to the setup shown in Figure 2? Such a comparison would provide a clear and direct evaluation of model performance prior to the SNV prioritization analysis in figure 3.
2. To demonstrate the necessity and contribution of the knowledge learned from Enformer, it would be valuable to include a benchmark model trained de novo without leveraging any pretrained foundation model—for example, a simple CNN mapping DNA sequence directly to tissue-specific epigenomic features. Otherwise, if a basic CNN achieves comparable performance, the advantage of utilizing Enformer as the foundation model would be questionable. Evaluating this comparison on a few representative transcription factors should be sufficient to illustrate the point.
3. For Figures 4C–E, could the authors include results from conventional TWAS analyses as a baseline comparison? Although some of these results are provided in the supplementary tables, researchers would likely be interested in directly visualizing how the SNVs derived from the TL models compare with results from standard TWAS approaches that use a larger set of SNPs.
4. Following the previous point, it would be helpful if the authors could include a discussion on the advantages and limitations of selecting SNVs from an external model when constructing TWAS linear models for gene expression prediction, compared to using the standard TWAS approach. Such a discussion could clarify the potential benefits of leveraging TL-derived SNVs, as well as any trade-offs or biases introduced by this selection strategy.

As a relevant example, but a different case, imagine a strategy in which half of the GWAS data are used to generate summary statistics for prioritizing the SNPs included in the TWAS model, and the remaining half are used for the gene–phenotype association test. What could happen?

5. In figure S2, the TL models seem not fully converged without reaching a plateau. Maybe the authors can elongate the training a bit. This is a minor and trivial issue.
6. It would be helpful to provide the complete list of transcription factors (TFs) associated with the diseases of interest, such as breast or prostate cancer, along with the subset of TFs used in the TF model training. Ideally, all relevant TFs would be included in the training; however, data availability may be a limiting factor. Providing these lists would allow users to better understand the potential limitations of the model and its coverage of disease-relevant regulatory factors.
7. The x-axis labels in Figure 3A–B (e.g., “1M”) should be centered between the two columns, as shown in Figure 4C–D.
