# Supplementary material for: Tissue-specific transfer learning improves functional variant and therapeutic target discoveries in breast and prostate cancer
Source: PLoS Genet. 2026 May 6;22(5):e1012145. doi: 10.1371/journal.pgen.1012145 (PMC13175468; doi:10.1371/journal.pgen.1012145)
Supplement: Attachment — Submitted filename: Responses_Letter.docx [file pgen.1012145.s025.docx]

**Reviewer #3**:

**R3.1** For comment3, the response from the reviewers "as average correlations around ~0.4 are well within the expected range for TF ChIP-seq prediction and align with previously published deep-learning models." However, why using TF ChIP-seq for prediction is the first question of concern. If prediction only ~0.4, how to expect predicted TF under ref and alt alleles are confident, and how the difference is confident to evaluate variant effect? As ChIP-seq is usually measured by binding sites (binary), not sure if what is predicted ChIP-seq track in a continuous manner here? Moreover, other deep learning models report on binary peak prediction for evaluating variant effect. More explanation and investigation towards this direction would be helpful.

**Responses:** We thank the reviewer for these thoughtful and important questions. Below, we clarify (i) why TF ChIP-seq signals were selected, (ii) why continuous-valued ChIP-seq profiles are appropriate, (iii) why mean correlations around ~0.4 remain acceptable for variant prioritization, and (iv) how ChIP-seq quality metrics relate to prediction performance.

**(i) Rationale for using TF ChIP-seq signals in transfer learning.** Transcription factors (TFs) are central regulators of gene expression, and genetic variants that alter TF binding frequently mediate cancer susceptibility. Rather than including all available TFs, we specifically selected those with demonstrated relevance to cancer biology: 18 breast cancer–associated TFs and 17 prostate cancer-associated TFs identified in our prior studies [PMID: 36402776; PMID: 34518541]. These TFs are enriched for cancer risk and functionally implicated in tumorigenesis. Fine-tuning Enformer on their corresponding ChIP-seq tracks enables the model to learn regulatory mechanisms active in cancer contexts, thereby improving functional variant prioritization and cancer-relevant gene discovery.

**(ii) Modeling TF ChIP-seq as continuous rather than binary signals.** Although TF binding is often described as binary (bound/unbound), ChIP-seq experiments generate continuous quantitative coverage profiles (bigWig files) that reflect read depth across genomic loci. Modeling these signals as continuous values preserves binding-strength information and provides richer quantitative resolution compared to binary peak labels. Continuous TF signal modeling has been consistently adopted in state-of-the-art regulatory genomics models, including Basenji [PMID: 32687525], Enformer [PMID: 34608324], and AlphaGenome [PMID: 41606153]. Following established practice, we processed TF ChIP-seq tracks by clipping extreme values at 32, scaling by 2 for numerical stability, and binning signals into 128-bp windows, consistent with Enformer’s Supplementary Table S2. These preprocessing details have been added to the **Data Preparation** section of **Materials and Methods**.

**Materials and Methods**

*“To mitigate large-scale discrepancies across tracks, output targets are normalized and clipped to a maximum value of 32, scaled by 2 (for numerical stability), and averaged across 128 positions to yield predictions at 128-bp resolution, resulting in 896 values (196608-40960*2)/128, 128 bp per bin) per trac. These preprocessing parameters were selected to be consistent with the TF ChIP-seq processing strategy described in Enformer’s Supplementary Table S2. Continuous TF ChIP-seq signals were used because they preserve binding-strength information, provide richer quantitative resolution, and is more informative for deep learning models than sparse binary peak labels.” (page 16)*

**(iii) Why mean Pearson correlations around ~0.4 are acceptable.** Predicting TF ChIP-seq signals is inherently challenging due to sparse TF occupancy, context-specific binding patterns, cell-line heterogeneity, and experimental noise. Benchmark models such as Basenji report TF ChIP-seq correlations ranging from 0.38 to 0.78. Our results span a comparable range (0.10-0.75 for breast; 0.08-0.82 for prostate), with mean correlations of approximately 0.42 (TL-breast) and 0.47 (TL-prostate), placing our performance within established expectations. The selection of dataset may contribute to our slightly lower correlations, as detailed in **(iv)** below. For TFs shared with Basenji, our performance is comparable or superior, for example, JUND (0.58 vs. 0.54) and POLR2A (0.78 vs. 0.66). We acknowledge that moderate global correlations may influence variant-effect estimation. However, our framework prioritizes rank ordering of variants rather than absolute signal magnitude. Because variant prioritization depends on consistent relative differences between alternative and reference allele predictions, moderate global correlations remain acceptable. Furthermore, our tCRA score aggregates allele-specific effects across multiple TFs, improving robustness. Downstream analyses, including enhancer enrichment, TWAS improvements, and gene-level associations, demonstrate that SNP-set prioritized genes are more functionally essential, therapeutically tractable, and disease-relevant, validating the biological relevance of our transfer learning framework.

**(iv)** **ChIP-seq tracks quality metrics and their prediction performance.** We observed substantial track-level variability for certain TFs, particularly ESR1 (ranging ~0.1–0.7) and AR (ranging ~0.08–0.6). Such variability likely reflects biological heterogeneity across cell lines and experimental platforms rather than architectural limitations of the model. Although TF ChIP-seq tracks were selected using Cistrome-recommended quality-control thresholds, we recognize that criteria suitable for general data quality assurance may not be sufficiently stringent for transfer learning, which involves limited fine-tuning and may therefore be more sensitive to track heterogeneity.

To investigate whether standard ChIP-seq quality metrics are associated with prediction performance, we stratified tracks into three correlation-based groups using Pearson r values computed across 1,937 held-out sequences (**S1-S2 Table**): low (r < 0.3), moderate (0.3 ≤ r ≤ 0.6), and high (r > 0.6). For breast, 275 tracks were distributed into 71 (low), 167 (moderate), and 37 (high); for prostate, 357 tracks were distributed into 40 (low), 250 (moderate), and 67 (high).

We evaluated six quality-control metrics across three group pairs (low-mod, low-high, mod-high): (1) FastQC score, (2) uniquely mapped read ratio, (3) PCR bottleneck coefficient, (4) PeaksFoldChangeAbove10, (5) fraction of reads in peaks (FRiP), and (6) union DNase I hypersensitive (DHS) ratio. Groups differences were assessed using both mean comparisons and Kolmogorov–Smirnov (KS) tests.

For breast tracks, the first three metrics showed no substantial differences across correlation groups (**S3A-C Fig**). In contrast, PeaksFoldChangeAbove10, FRiP, and union DHS ratio displayed significant distributional differences between the low-correlation group and both moderate and high groups (**S3D-F Fig**). For example, FRiP showed highly significant differences between low vs. moderate (P = 2 x 10⁻¹¹) and low vs. high (P = 6 x 10⁻⁹), whereas moderate vs. high was not significant (P = 1 x 10⁻²). Similar trends were observed for PeaksFoldChangeAbove10 and union DHS ratio.

For prostate tracks, the first three metrics again showed no significant differences (**S4A-C Fig**), whereas PeaksFoldChangeAbove10 and FRiP differed significantly between the low-correlation group and the other groups (**S4D-E Fig**).

Collectively, these results indicate that lower Pearson correlation tracks are associated with greater heterogeneity in peak-enrichment metrics, particularly FRiP and PeaksFoldChangeAbove10. These findings suggest that applying more stringent thresholds (e.g., PeaksFoldChangeAbove10 ≥ 1000; FRiP > 0.02) may maintain high-quality tracks which suitable for transfer learning applications.

We have updated the **Results, Materials and Methods,** and **Discussion** sections to reflect these analyses.

**Results**

*“In addition, we observed considerable variability in prediction performance across TF ChIP-seq tracks, particularly for ESR1 (r = 0.10-0.75) and AR (r = 0.08-0.60), suggesting heterogeneity across cell lines or experimental platforms. We stratified tracks into three correlation-based groups (low, moderate, and high) and evaluated their associations with standard ChIP-seq quality metrics (Materials and Methods). For breast, 275 tracks were distributed into 71 (low), 167 (moderate), and 37 (high). For prostate, 357 tracks were distributed into 40 (low), 250 (moderate), and 67 (high). In both datasets, general sequencing metrics (FastQC score, uniquely mapped read ratio, and PCR bottleneck coefficient) did not differ substantially across performance groups (****S3A-C Fig, S4A-C Fig****). In contrast, peak-enrichment metrics (PeaksFoldChangeAbove10, FRiP, and union DHS ratio) were significantly lower in low-correlation tracks compared to moderate and high groups in breast (****S3D-F Fig****), with similar trends observed in prostate (****S4D-E Fig****).” (page 7)*

**S3 Fig: Box plots of ChIP-seq quality metrics stratified by Pearson correlation performance (low, moderate, and high) across 257 breast tissue tracks. A)** FastQC score, reflecting overall sequencing quality. **B)** Uniquely mapped read ratio, representing the proportion of reads that align uniquely to the reference genome. **C)** PCR bottleneck coefficient (PBC), measuring library complexity and PCR duplication bias. **D)** Peaks Fold Change over 10, indicating the proportion of peaks with at least ten-fold enrichment over background signal, reflecting peak strength. **E)** Fraction of reads in peaks (FRiP), quantifying signal enrichment within called peak regions. **F)** Union DNase I hypersensitive site (DHS) ratio, representing the fraction of reads overlapping open chromatin regions.

**S4 Fig: Box plots of ChIP-seq quality metrics stratified by Pearson correlation performance (low, moderate, and high) across 375 prostate tissue tracks. A)** FastQC score. **B)** Uniquely mapped read ratio. **C)** PCR bottleneck coefficient (PBC). **D)** Peaks Fold Change over 10. **E)** Fraction of reads in peaks (FRiP). **F)** Union DNase I hypersensitive site (DHS) ratio.

**Materials and Methods**

*“****Evaluation of ChIP-seq tracks quality metrics and their prediction performance.*** *Based ont the Pearson correlation coefficient performance, tracks were stratified into three groups: low (r < 0.3), moderate (0.3 ≤ r ≤ 0.6), and high (r > 0.6). To assess whether ChIP-seq quality metrics were associated with prediction performance, we compared the distributions of six Cistrome-reported quality metrics (FastQC score, uniquely mapped read ratio, PCR bottleneck coefficient, PeaksFoldChangeAbove10, fraction of reads in peaks (FRiP), and union DNase I hypersensitive (DHS) ratio) across the three groups. Pairwise differences (low vs. moderate, low vs. high, moderate vs. high) were evaluated using the Kolmogorov–Smirnov (KS) test. We considered a significant difference (KS P < 1 × 10⁻⁵) as evidence that the corresponding quality metric is associated with differences in prediction performance between groups.” (page 18)*

**Discussion**

*“Third, the substantial variability in Pearson correlation observed for ESR1 and AR likely reflects biological and technical heterogeneity rather than model limitations. Notably, low-correlation tracks displayed reduced peak-enrichment metrics (PeaksFoldChangeAbove10 and FRiP) compared to moderate and high groups, underscoring the importance of peak-strength measures in track selection. These findings suggest that transfer learning, given its limited fine-tuning steps, may require more stringent quality thresholds and careful curation of ChIP-seq tracks than conventional training approaches.” (page 15)*
